# Persistent mRNA and miRNA expression changes in irradiated baboons

**DOI:** 10.1038/s41598-018-33544-2

**Published:** 2018-10-18

**Authors:** Matthias Port, Francis Hérodin, Marco Valente, Michel Drouet, Patrick Ostheim, Matthäus Majewski, Michael Abend

**Affiliations:** 1 Bundeswehr Institute of Radiobiology, Neuherbergstr. 11, Munich, 80937 Germany; 2 Institut de Recherche Biomedicale des Armees, Bretigny-sur-Orge, 91220 France

## Abstract

We examined the transcriptome/post-transcriptome for persistent gene expression changes after radiation exposure in a baboon model. Eighteen baboons were irradiated with a whole body equivalent dose of 2.5 or 5 Gy. Blood samples were taken before, 7, 28 and 75–106 days after radiation exposure. Stage I was a whole genome screening for mRNA combined with a qRT-PCR platform for detection of 667 miRNAs. Candidate mRNAs and miRNAs differentially up- or down-regulated in stage I were chosen for validation in stage II using the remaining samples. Only 12 of 32 candidate genes provided analyzable results with two mRNAs showing significant 3–5-fold differences in gene expression over the reference (p < 0.0001). From 667 candidate miRNAs, 290 miRNA were eligible for analysis with 21 miRNAs independently validated using qRT-PCR. These miRNAs showed persistent expression changes on each day and over days 7–106 days after exposure (n = 7). In particular miR-212 involved in radiosensitivity and immune modulation appeared persistently and 48–77-fold up-regulated over the entire time period. We are finally trying to put our results into a context of clinical implications and provide possible hints on underlying molecular mechanisms to be examined in future studies.

## Introduction

Taking advantage of a non-human primate model with high genetic homology to humans, we wondered whether radiation exposure in healthy animals might result in gene expression changes in the peripheral blood persisting over weeks and months after radiation exposure. Clearly, those examinations cannot be performed in humans and in patients treated with radiation therapy it is impossible to discriminate whether changes in gene expression origin from the disease they are suffering from or the radiation exposure. A literature review identified two areas where persistent gene expression might be of clinical interest.

Patients treated for a first cancer are at risk for a second tumor after radiation or chemotherapy^1–4^. Small RNAs such as miRNAs are recognized to play an important role in cancer diagnosis and even therapy^5–9^. Regarding the classification of different tumor types, miRNAs might be better than protein coding mRNA^10^. For example, it was reported that a signature miRNA was downregulated 1–2 years prior to the diagnosis of lung cancer, suggesting the prediction potential using miRNAs^5^. Provided these changes in post-transcriptional miRNAs are causally related to tumor development, these could serve as therapeutic targets. Prerequisite of a sufficient diagnosis or prediction of the later occurring tumor entity are persistent radiation-related gene expression changes after exposure.

Radiation-related or cancer-associated behavioral symptoms such as fatigue, depression and sleep disturbances are reported after treatment of tumor entities such as breast cancer^11–13^, prostate cancer^14^ or lung cancers^15^. In particular fatigue is difficult to manage medically since no single effective intervention exists for severe fatigue other than for e.g. pain^16^. Fatigue is reported to last over months or even years after cancer therapy^15,17^. Inflammatory processes are reported to represent a possible mechanism for the fatigue^18,19^ as well as other mechanisms such as disrupted metabolism of adenosine triphosphate^16^. However, currently, there is no accepted pathophysiological evidence to explain the development of fatigue^16^ and it is challenging to discriminate whether fatigue is cancer- or radiation-related. Examinations of persistent gene expression changes might contribute to understanding long-term post-treatment sequelae and might provide clues about an underlying molecular basis for it.

In collaboration with the French Army Biomedical Research Institute, we assessed blood samples obtained from irradiated healthy baboons taken before (day 0), 7 days, 28 days and 75–106 days after partial or total body exposure. On a subset of the blood samples, we performed a whole genome screening and identified protein coding mRNA genes (screening stage I). These mRNAs were then validated (stage II) using qRT-PCR with the remaining samples. In stage I we also screened for 667 miRNAs using a qRT-PCR platform. The selected candidate miRNAs were validated on the remaining samples in stage II using the same qRT-PCR platform but restricting the analysis to the candidate miRNAs from stage I.

With this design we searched for persistent gene expression changes on the transcriptional (protein coding mRNAs) and the post-transcriptional level (miRNAs) so that we might add information on the significance of mRNA or miRNAs for cancer diagnosis. The irradiation of healthy baboons enables the identification of radiation-related gene expression changes which might represent the molecular basis for post-treatment fatigue and the discrimination from a cancer-related fatigue.

## Materials and Methods

### Animals and ethical approval

Eighteen baboons were bred by the Centre National de la Recherche Scientifique (Rousset sur Arc, France) for the purpose of biomedical research. In the nonhuman primate facility of the French Army Biomedical Research Institute, the baboons were placed in individual cages at 21 °C, with a relative humidity of 55% and a 12 h/12 h light-dark schedule. The animals received fresh fruit and solid food twice a day, and had access to water *ad libitum*. The male baboons had an average age of 8.1 years (±3.3 years) and weighed 23.7 (±5.2 kg). The experiment was approved by the French Army Animal Ethics Committee (No 2010/12.0). All baboons were treated in compliance with the European legislation related to animal care and protection in order to minimize discomfort. All applicable international, national and institutional guidelines for the care and use of animals were followed. All procedures performed in studies involving animals were in accordance with the ethical standards of the institution or practice at which the studies were conducted.

### Irradiation

The animals were anesthetized with a combination of tiletamine and zolazepam (6 mg.kg^−1^ intramuscularly, Zoletil 100; Virbac, Carros, France) before irradiation. Then, the baboons were placed in restraint chairs, sitting orthogonally, front to a horizontal and homogeneous field of gamma rays delivered by a ^60^Co source (IRDI 4000; Alsthom, Levallois, France) to perform either total body irradiation (TBI) or partial body irradiation (PBI). In order to attain different patterns of PBI, a 20 cm thick lead screen was used to shield different parts of the body (for details see^20,21^. Two baboons were exposed to 5 Gy TBI and two others to 2.5 Gy TBI. Eight different exposure patterns were simulated and two baboons were exposed per pattern which summed up to 14 baboons receiving PBI (for details see^20,21^) corresponding to an equivalent TBI dose of 2.5 or 5 Gy. Two dose rates were used (8 cGy/min for 5 Gy TBI and 5 Gy 50% PBI, and 32 cGy/min for all other situations) because the Cobalt 60 source was changed during this study. Moreover, to achieve the same homogeneous radiation field whatever the dose rate, all baboons were irradiated at the same distance from the source. Consequently, radiation exposures lasted between 8 min and 62 min. The mid-line tissue (right anterior iliac crest) dose in air was measured with an ionization chamber. Delivered doses were monitored by alumina powder thermoluminescent dosimeters placed on different cutaneous areas (thorax, thoracic and lumbar vertebrae, head, tibia, femur, femoral head, for details see^20,21^).

A full-time attending veterinarian (AV) was employed in the nonhuman primate facility. A written program of veterinary care was applied with regularly scheduled visits. The AV monitored the baboons during the whole study. Specifically, the veterinary surgeon in charge of animal welfare fulfilled a mission of council and inspection to ensure that nonhuman primates were provided supportive care in ways that minimize fear, pain, stress, and suffering. Accordingly, two baboons showing signs of pain received buprenorphine (Vetergesic Multidose, Sogeval, Sheriff Hutton, UK). A transient weight loss of 4–10% was observed in 3 baboons, but in general no change of behaviour and activity was observed and animals moved in their cages as usually.

### RNA extraction and quality control

Whole blood samples (2.5 ml) were processed following the PAXgene Blood RNA system (BD Diagnostics, PreAnalytiX GmbH, Hombrechtikon, Switzerland). In brief, blood was drawn into a PAXgene Blood RNA tube at the French Army Biomedical Research Institute. The tube was gently inverted (10 times), stored at room temperature overnight then at −20 °C. After all samples were collected, the PAXGene tubes were sent to Germany for further processing. After thawing, washing and centrifugation, cells in the supernatant were lysed (Proteinase K, PAXgene Blood RNA protocol) followed by addition of Lysis/Binding Solution taken from the mirVana kit (Life Technologies, Darmstadt, Germany). With the mirVana kit total RNA, including small RNA species, was isolated by combining a Phenol-Chloroform RNA precipitation with further processing using a silica membrane. After several washing procedures, DNA residuals became digested on the membrane (RNAse free DNAse Set, Qiagen, Hilden, Germany). RNA was eluted in a collection tube and frozen at −20 °C. Quality and quantity of isolated total RNA were measured spectrophotometrically (NanoDrop, PeqLab Biotechnology, Erlangen, Germany). RNA integrity was assessed by the 2100 Agilent Bioanalyzer (Life Science Group, Penzberg, Germany) and DNA contamination was controlled by conventional PCR using an actin primer. We used only RNA specimens with a ratio of A_260_/A_280_ ≥ 2.0 (Nanodrop) and RNA integrity number (RIN) ≥7.5 for whole genome microarray (IMGM Laboratories, Martinsried, Germany) or RIN ≥7.3 for qRT-PCR analyses.

### Stage I screening for mRNAs and miRNAs

The whole genome screening for differentially expressed genes (DEG) (protein coding mRNAs) was performed on 26 RNA samples taken from about one third of the baboons for each of the four time points which was before (pre-exposure samples, n = 5), 7 days (n = 7), 28 days (n = 7) and 75–106 days (n = 7) after radiation exposure. In order to increase the robustness we used pre-exposure RNA samples from other baboons than the RNA samples taken after irradiation (Table 1). The sample number for screening purposes taken after irradiation was higher (seven instead of five), because of their higher yield of RNA per sample, which were not used in previous examinations and, therefore, more RNA remained for this analysis on persistent gene expression over time. We used the Agilent oligo microarray GE 8 × 60 K (Agilent Technologies, Waldbronn, Germany) combined with a one-color based hybridization protocol of GeneSpring GX12 software for data analysis as described in detail elsewhere^22^. We analyzed gene expression by quantile normalized log_2_-transformed probe signals as an outcome. We used the non-parametric Mann Whitney (MW) test to compare gene expression across the different time points using the unexposed group as the reference (control). Only those gene transcripts that had a call “present” in at least 60% of RNA specimens were included in the analysis of gene expression and only genes with MW p-values ≤0.05 and a ≥2-fold gene expression difference among compared groups were considered to represent a candidate gene for validation in stage II. In parallel, a commercially available 384-well low density arrays (LDA) high throughput qRT-PCR platform was used that provided the simultaneous detection of 380 different miRNAs. Two different LDAs (type A and B) were combined so that the detection of 667 miRNA species (partly spotted in duplicate to completely fill the LDA) was possible. For miRNA screening purposes we used the same samples as used for the mRNAs. Due to the explorative nature of this study, the low sample size and the nonparametric statistics employed, we did not correct for multiple comparisons on the screening stage I of the study, but considered this within the validation stage II of our study where the numbers of hypotheses tested in parallel was reduced from about 20,000 (stage I) to 32 mRNAs and 21 miRNAs in stage II (see below). Gene expression data presented in this publication have been deposited at the NCBI’s Gene Expression Omnibus (GEO accession number GSE77254).

**Table 1:** The left part of the table describes exposure details of animals irradiated following different partial body irradiation (PBI) exposure pattern or total body irradiation (TBI) exposures with different doses resulting in each animal in either 2.5 Gy or 5 Gy whole body equivalent doses.

ID #	Exposure (Co-60)			days after exposure	stage I screening, samples from days…	stage II independent validation, samples from days…
	radiation dose (Gy, free in air)/ body area (%)	PBI	TBI			
1	5 PBI 50%	left-hemibody exposed		0	0	
				7		7
				28		28
				75–106		75–106
2	5 PBI 50%	left-hemibody exposed		0		0
				7	7	
				28	28	
				75–106	75–106	
3	15 PBI 30%	head + arms exposed		0		0
				7	7	
				28	28	
				75–106	75–106	
4	15 PBI 30%	head + arms exposed		0		0
				7	7	
				28	28	
				75–106	75–106	
5	6.25 PBI 80%	2 legs shielded		0		0
				7		7
				28		28
				75–106		75–106
6	6.25 PBI 80%	2 legs shielded		0		0
				7	7	
				28	28	
				75–106	75–106	
7	10 PBI 50%	left-hemibody exposed		0		0
				7		7
				28		28
				75–106		75–106
8	10 PBI 50%	left-hemibody exposed		0		0
				7	7	
				28	28	
				75–106	75–106	
9	5.55 PBI 90%	1 leg shielded		0		0
				7	7	
				28	28	
				75–106	75–106	
10	5.55 PBI 90%	1 leg shielded		0		0
				7		7
				28		28
				75–106		75–106
11	6.25 PBI 80%	2 legs shielded		0	0	
				7		7
				28		28
				75–106		75–106
12	6.25 PBI 80%	2 legs shielded		0		0
				7		7
				28		28
				75–106		75–106
13	2.5 TBI 100%		TBI	0	0	
				7		7
				28		28
				75–106		75–106
14	2.5 TBI 100%		TBI	0		0
				7	7	
				28	28	
				75–106	75–106	
15	5 TBI 100%		TBI	0	0	
				7		7
				28		28
				75–106		75–106
16	5 TBI 100%		TBI	0	0	
				7		7
				28		28
				75–106		75–106
17	7.5 / 2.5 TBI 100%		TBI	0		0
				7		7
				28		28
				75–106		75–106

The right part of the table depicts the assignment of RNA samples to the screening (stage I) or the independent validation (stage II) phase of the study.

### Stage II: Validation of stage I candidate genes via qRT-PCR

For validating the mRNA candidate genes from stage I (screening) using remaining RNA samples, we used a custom LDA and TaqMan chemistry (order IDs for the examined genes are provided in Table 2). A 1 µg RNA aliquot of each RNA sample was reverse transcribed using a two-step PCR protocol (High Capacity Kit). 400 µl cDNA (1 µg RNA equivalent) was mixed with 400 µl 2 x RT-PCR master mix and pipetted into the 8 fill ports of the LDA. Cards were centrifuged twice (1,200 rpm, 1 min, Multifuge3S-R, Heraeus, Germany), sealed, and transferred into the 7900 qRT-PCR instrument. The qRT-PCR was run for two hours following the qRT-PCR protocol for 384-well LDA format. All measurements were run in duplicate.

**Table 2:** The left part of the table summarizes results from the screening stage for differentially expressed mRNAs over time using microarrays.

screening with microarray						validation with qRT-PCR							agreement screening/validation
probe name	Gene Symbol	Description	mean differential gene expression over time (d)			TaqMan Assay ID	mean diff. gene expression over time (d)			p-values per day			
			7	28	75–106		7	28	75–106	7	28	75–106	
A_33_P3514487	VSTM1	V-set and transmembrane domain containing 1	0,2	0,3	0,2	Hs00419073_m1							
A_32_P524614	C17orf74	chromosome 17 open reading frame 74	0,3	0,3	0,3	Hs00545612_s1							
A_23_P72584	ACBD7	acyl-CoA binding domain containing 7	0,3	0,3	0,2	Hs00744676_s1							
A_33_P3341787	IDS	iduronate 2-sulfatase	0,4	0,5	0,5	Hs00240821_m1	0,9	0,6	1,3	0,3	0,1	0,2	not
A_33_P3671378	CERCAM	cerebral endothelial cell adhesion molecule	0,4	0,4	0,3	Hs00170969_m1							
A_23_P251043	SYNDIG1	synapse differentiation inducing 1	0,3	**0**,**3**		Hs00227616_m1	1,1	**0**,**3**	1,1	0,8	**0**,**04**	0,7	partly
A_33_P3285734	FCRL6	Fc receptor-like 6	0,3	0,4		Hs02341772_m1	0,6	0,5	1,2	0,3	0,4	0,6	not
A_24_P162373	ZNRF3	zinc and ring finger 3	0,4	0,4		Hs00393094_m1							
A_23_P8913	CA2	carbonic anhydrase II	**4**,**7**	**3**,**0**		Hs01070108_m1	**2**,**2**	**3**,**1**	1,0	**0**,**07**	**<0**.**0001**	0,9	complete
A_23_P37994	TP53TG3	TP53 target 3	4,0	2,7		Hs00414226_g1							
A_23_P204016	CACNB3	calcium channel, voltage-dependent, beta 3 subunit	3,5	3,2		Hs00893804_g1	0,3	0,4	1,1	**0**,**002**	**0**,**02**	0,74	inverse
A_33_P3249259	TGM6	transglutaminase 6	3,1	2,2		Hs00975389_m1							
A_33_P3815935	TMEM50B	RST40456 Athersys RAGE Library	2,9	2,4		Hs00272046_m1	0,8	0,5	1,3	0,6	**0**,**04**	0,2	inverse
A_32_P147078	SLC8A1	solute carrier family 8 (sodium/calcium exchanger), member 1	2,8	2,5		Hs00253432_m1							
A_23_P351837	KLHL35	kelch-like 35	2,7	2,6		Hs00400533_m1							
A_19_P00805942	SLC7A14	solute carrier family 7, member 14	2,7	2,2		Hs00703486_s1							
A_33_P3213512	COQ5	coenzyme Q5 homolog, methyltransferase	2,7	2,2		Hs00260456_m1		0,7	1,0		0,1	0,9	not
A_23_P17811	SEC. 14L2	SEC. 14-like 2	2,6	2,1		Hs01052193_g1							
A_23_P60517	FXN	frataxin (FXN), nuclear gene encoding mitochondrial protein	2,6	2,7		Hs00175940_m1							
A_23_P166526	RIBC2	RIB43A domain with coiled-coils 2	2,5	2,5		Hs00210331_m1							
A_23_P75038	DCLRE1A	DNA cross-link repair 1 A	2,3	2,6		Hs00384872_m1							
A_33_P3294017	HNRNPC	heterogeneous nuclear ribonucleoprotein C (C1/C2)		3,7	3,8	Hs01028910_g1	0,8	0,3	1,0	0,2	**0**,**0095**	0,9	inverse
A_23_P251647	RFESD	Rieske (Fe-S) domain containing		2,7	2,1	Hs00379296_m1							
A_33_P3260605	CTNNAL1	catenin (cadherin-associated protein), alpha-like 1		2,7	2,2	Hs00169384_m1							
A_24_P346126	SERF1A	small EDRK-rich factor 1A (telomeric)		2,6	2,6	Hs00743093_s1							
A_33_P3229122	HIST1H2BF	histone cluster 1, H2bf		2,3	2,1	Hs00371409_s1							
A_33_P3256303	SELPLG	selectin P ligand		**0**,**3**	0,5	Hs00380945_m1	1,1	**0**,**4**	1,1	0,8	**0**,**02**	0,6	partly
A_23_P50919	SERPINE2	serpin peptidase inhibitor, clade E		0,4	0,4	Hs00299953_m1							
A_33_P3461633	LOC284454	uncharacterized LOC284454, non-coding RNA		0,5	**0**,**3**	Hs01065212_s1	0,4	**0**,**2**	1,2	0,3	**0**,**006**	0,7	partly
A_24_P228550	TUBB1	tubulin, beta 1 class VI		0,5	0,3	Hs00258236_m1	1,6	0,6	1,0	0,3	0,2	0,9	not
A_23_P47410	ESAM	endothelial cell adhesion molecule		0,5	0,4	Hs00332781_m1	2,2	**0**,**6**	1,0	**0**,**04**	0,2	0,8	partly
A_23_P74114	ZNF713	zinc finger protein 713		0,5	0,4	Hs00403582_m1							

The right part of the table shows the results from the validation stage. In total 32 candidate genes from screening were validated employing a specific primer-probe design (TaqMan Assay ID). Differential gene expression (fold changes relative to unexposed controls) and corresponding p-values are shown per day for each mRNAs where amplification plots were developed in ≥60% of the examined samples. Fold-changes in gene expression and associated p-values that showed successful validation are shown in bold letters. The last column summarizes the agreement between screening and validation results. A complete agreement reflects an up- or down-regulation in the same direction in both stages over e.g. several days and a partial agreement refers to a validation of e.g. only changes in gene expression of one and not all days differentially expressed during the screening stage. An inverse agreement represents significant changes in gene expression in one direction (e.g. up-regulated) during the screening and an opposite significant regulation (e.g. down-regulated) during the validation stage.

Two commercially available 384-well LDAs (type A and B) were used for detection of 667 miRNA species in stage I (see above), but only those candidate miRNAs showing significant changes in miRNA expression over the unexposed group were independently validated in stage II. Aliquots from each RNA sample (in general 2 µg total RNA/LDA type A/B) were reversely transcribed without preamplification over three hours using *“Megaplex pools without preamplification l for microRNA expression analysis protocol”*. Using different sets of primers, two kinds of cDNAs suitable for each of both LDAs were created. In a second step, the whole template cDNA and 450 µl 2x RT-PCR master mix were adjusted to a total volume of 900 µl by adding nuclease free water, and aliquots of 100 µl were pipetted into each fill port of a 384-well human LDA. Cards were centrifuged twice (see above), sealed, transferred into the 7900 RTQ-PCR instrument and again the 384-well LDA RTQ-PCR protocol was run over two hours.

All technical procedures for qRT-PCR were performed in accordance with standard operating procedures implemented in our laboratory in 2008 when the Bundeswehr Institute of Radiobiology became certified according to DIN EN ISO 9001/2008. All chemicals for qRT-PCR using TaqMan chemistry were provided by Life Technologies, Darmstadt, Germany.

For the custom LDA, threshold cycle (CT) values were normalized relative to the 18S rRNA measured in an aliquot of the RNA samples using a 96-well-format TaqMan qRT-PCR platform. We have found that this approach to normalization was more robust compared to the use of the internal control (GAPDH and 18 S rRNA) spotted on the LDA. For the commercial miRNA LDAs type A and B we used the median miRNA expression on each LDA for normalization purposes, because this proved to be more robust and a slightly more precise method compared to a normalization approach using a housekeeping miRNA species provided on the LDA (data not shown). The CT-values of the housekeeping gene was subtracted from the CT-value of each of the spotted genes, following the ∆CT-quantitative approach for normalization purposes.

### Statistical analysis

During screening for potential candidate mRNAs or miRNAs for persistent changes in gene expression over time we used the same biosamples consisting of 5–7 samples per time point and used the unexposed pre-exposure samples as the reference. The filter (criteria) that we used was statistically significant (p ≤ 0.05) and ≥2-fold mRNA and miRNA expression changes over time after radiation exposure in those RNA species that had a call “present” in at least 60% of the samples. The validation was performed using the qRT-PCR gene expression results. During validation we assessed the assumptions of normality (Kolmogorov-Smirnov) and equal variance and if required utilized either the pooled (equal variance) or the Satterthwaite variant of the t-test (unequal variance). Normality of mRNA and miRNA expression values was achieved after log transformation where necessary. For these RNA species we used a Bonferroni correction to adjust the p-values for multiple comparisons. Descriptive statistics (n, mean, standard deviation, min, max) and p-values (t-test and the nonparametric Kruskal-Wallis (KW) test, where applicable) were calculated for each of the RNA species and per time point. After the validation we performed logistic regression analysis for each of the variables (genes) of interest separately (univariate) and using all samples together to increase the power. Odds ratios (OR), 95% confidence intervals (95% CI) and corresponding p-values (Wald Chi-Square) were calculated. We also determined the area under a receiver-operator characteristic (ROC) curve providing a reasonable indication of overall diagnostic accuracy. ROC areas of 1.0 indicate complete distinction between pre-exposure samples and time points after exposure. All calculations were performed using SAS (release 9.2, Cary NC, USA).

## Results

### Stage I: screening for candidate mRNAs and miRNAs

On average, we isolated 10 (stdev +/−2.9), 5.3 (+/−3.7), 13.4 (+/−9.3) and 8.1 (+/−2.2) µg total RNA from 2.5 ml whole blood before irradiation and 7, 28 and 75–106 days after irradiation, respectively. RNA integrity (RIN) with mean values of 8.5 (stdev +/−0.5), 9.0 (+/−0.5) 9.0 (+/−0.3) and 8.6 (+/−0.5) on these time points suggested high quality RNA sufficient for running both methods.

**Figure 1:**
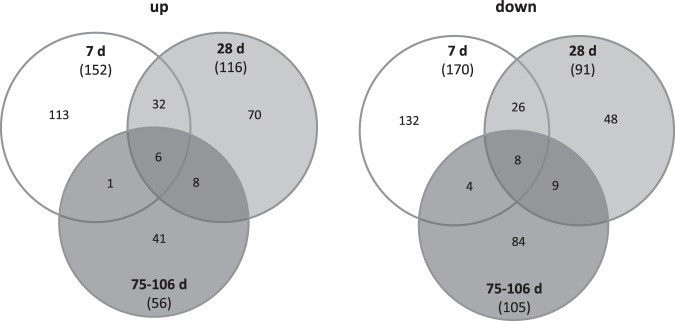
Venn diagrams showing the number of up- (left side) and down-regulated (right side) protein coding genes (mRNA transcripts) observed at 7, 28 and 75–106 days after radiation exposure. The number of differentially expressed genes (DEG) observed on one, two or all three time points after exposure is shown in the corresponding overlapping circles. Numbers in parenthesis represent the total number of differentially expressed genes.

From about 20,000 protein coding mRNAs, 46% on average over all time points (range: 41–49%) appeared expressed. An about equal number of 324 up-regulated and 366 down-regulated DEG was observed on days 7–106 after irradiation, but less than 10 genes showed an overlap in up- or down-regulated gene expression changes over all time points (Fig. 1). Also, a 2–3-fold reduction in the number of DEG was observed over time (Fig. 1).

Based on the fold-difference, the p-value and sustained changed mRNA expression over the days after irradiation, we selected 32 candidate mRNAs for validation at stage II (Table 2, left part).

During the screening of 667 miRNAs, we identified 290 miRNAs eligible for analysis. In total, 70 miRNAs revealed significant expression changes after radiation exposure and were forwarded for validation in stage II (data not shown).

### Stage II: validation using qRT-PCR measurements

**Figure 2:**
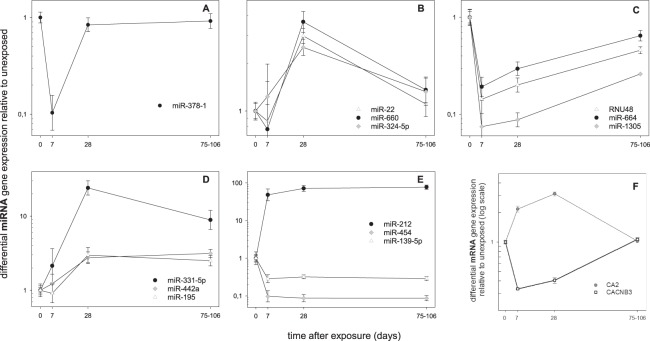
Differential gene expression changes over time are depicted for miRNAs (**A–E**) and mRNAs (**F**). Up to three examples for genes either differentially expressed only on days 7 (**A**) or 28 (**B**) and on two consecutive days (7–28, (**C**) or 28–75, (**D**) or over the whole period of time (**E**) are shown. Candidate mRNAs differentially expressed on days 7–28 are shown in panel **F**. Symbols represent geometric mean values and error bars reflect the standard error of mean. Numbers of measurements per group are shown in Supplement Table 2.

During stage II, validation of the 32 candidate mRNAs from stage I, we found 20 mRNAs showed either no amplification plot or amplification plots in a minority of all samples (n ≤ 3). Those were excluded from further analysis. There remained 12 genes for validation, but a validation of gene expression changes in the same direction as seen during stage I screening was either not detected (n = 4), or only in a part of the indicated time points after irradiation (n = 4, Table 2, right side). Another three mRNAs showed an inverse regulation in gene expression among the screening and the validation samples. For instance, during stage I screening, *CACNB3* appeared about 3-fold up-regulated on days 7 and 28 after exposure, but during validation at stage II a statistically significant (p = 0.002) 3-fold down-regulation of the remaining samples using qRT-PCR was observed (Table 2, Fig. 2F). Only *CA2* showed a 2–5-fold up-regulation on days 7 and 28 during screening and validation with a borderline significance at day 7 (p = 0.07) and a high significance at day 28 (p < 0.0001, Table 2, Fig. 2F).

Independent validation of the 70 miRNAs from the screening stage resulted in 21 miRNAs which were significantly differentially expressed only on days 7 (n = 1, Fig. 2A) or 28 (n = 4, Fig. 2B) or over two time points such as 7 and 28 (n = 4, Fig. 2C) or 28 and 75–106 days after irradiation (n = 7, Fig. 2D, Supplement Table 2). Another five miRNAs revealed persistent DEG at all three time points (Fig. 2E, Supplement Table 2). For these 21 miRNAs, all data from stage I and stage II were merged and analyzed together resulting in highly significant DEG that survived Bonferroni correction for multiple comparisons (p < 0.002 [0.05/21]) and ROC between 0.9–1.0 were calculated for most genes suggesting a complete or almost complete separation of gene expression relative to the pre-exposure reference (bold p-values, Supplement Table 2, right part). A 2-6-fold up- or downregulation was observed in 17 of the 21 miRNAs, but 10-28-fold (e.g. miR-378-1, miR-1305, miR-331-5p, miR454) or even up to 77-fold changes in miRNA expression (miR-212) were observed in the remaining 5 miRNAs. In particular miR212 revealed a persistent 48-77-fold DEG over all three time points after irradiation (Supplement Table 2, Fig. 2E**)**.

## Discussion

Taking advantage of a non-human primate model with high genetic homology to humans, we examined the whole genome for radiation-related transcriptional (protein coding mRNAs) and post-transcriptional (non-coding miRNAs) gene expression changes in the peripheral blood persisting over weeks and months after radiation exposure. At this time frame after exposure, the baboons’ peripheral blood had recovered from the radiation effects and showed normal blood cell counts. In other words, does radiation cause a long lasting altered transcriptome/post-transcriptome in previously irradiated individuals? This examination was also challenged by employing different partial and whole body exposure patterns in order to scope with different exposure scenarios. However, it was the goal of this research to examine persistent changes in gene expression which could be found independently of partial and whole body exposure patterns resulting in either 2.5 Gy or 5 Gy whole body equivalent doses. Examinations such as that cannot be performed in healthy humans, but might provide insights into radiation effects occurring delayed after exposure (see below).

During the transcriptome screening, several hundred up- and down-regulated mRNAs were identified with 14 mRNAs consistently differing from the reference for the three time points after exposure. Due to methodological reasons, most of the 32 selected mRNAs could not be validated, except for *CA2* which appeared 2-5-fold up-regulated during screening as well as the validation on the remaining samples (Fig. 2F). *CA2* (carbonic anhydrase 2) catalyzes reversible hydration of carbon dioxide. Defects in this enzyme are associated with e.g. osteopetrosis (marble bone disease). Another gene, *CACNB3* (calcium voltage-gated channel auxiliary subunit beta 3, with a role in the regulation of transcription factors and calcium transport) appeared about 3-fold up-regulated during screening and about 3-fold down-regulated during validation (Fig. 2F). An inverse regulation during the validation process using another method and other biological samples is a very rare event, however the p-value (p = 0.002) supports the robustness of the findings and argues against a result by chance. Since the microarray targeted another exon than the one used for validation by the Taqman assay, it is more likely that exons of the same gene might show inverse gene expression values after radiation exposure when considering mechanisms such as alternative splicing and cassette exons^23^. So, the interpretation of some mRNA findings is challenging considering these methodological issues.

Regarding our post-transcriptomic analysis, we identified substantial changes in the expression of 21 successfully validated miRNAs (Fig. 2A–E). Some of these altered miRNAs showed a transient alteration e.g. observed only on the seventh or the 28^th^ day after exposure, but not later (Fig. 2A,B). However, we found that six miRNAs persisted over the whole period of time (Supplement Table 2, Fig. 2E). For instance, 48 to 77 fold-differences relative to unexposed blood samples taken before radiation exposure were observed for miR-212 and persisted over the entire time period. Another five miRNAs showed 6-10-fold differences and the remaining miRNAs showed 2-3-fold differences. All of this suggests large post-transcriptional miRNA changes that, to our knowledge, have not been previously reported in other radiation-models either *in vitro* or *in vivo*. ROC areas at one or close to one indicate a comparable response among the 17 radiation exposed baboons and with the accompanying low p-values reduce the likelihood of chance findings.

Again, these results indicate a marked radiation-related modification of the post-transcriptome likely occurring in irradiated cells (7 days after exposure) and certainly in the following cell generations up to 75–106 days after exposure. The cellular progeny represent cells that were not directly exposed to radiation. Because our measurements were performed on peripheral whole blood, presumably our results reflect either altered blood stem cells or indicate a selection for less radiosensitive stem cells that reconstitute the peripheral blood after radiation exposure. We wondered about the possible clinical implication of our findings which were not examined within this study and are, therefore, speculative. Interestingly, many of our miRNA species are already known to be linked with radiosensitivity (e.g. miR-22, miR-29c, miR-195, miR-212) or chemotherapy resistance (e.g. miR-331-5p, supplement Table 1). Also, most of these miRNAs are thought to be associated with risk of cancers to the colon, thyroid and lung or acute lymphoblastic or chronic myeloid leukemia (supplement Table 1). Tumorigenesis represents a multistep process. We speculate that this shift in the composition of cells might make the exposed individuals more prone for developing a tumor during their follow-up and as that might represent the first step towards tumorigenesis. It is by chance which of the animals might get the next hit and finally develop a tumor, but, certainly, radiation exposed individuals are under higher risk for developing secondary tumors and the changes observed might represent the first step for tumor initialization. Still, whether these persistent gene expression alterations are associated with risk of primary or secondary tumors is a hypothesis for future research. Further bioinformatic analysis on our over time persistently deregulated miRNAs using the DIANA-miRPath 3.0 tool (search for categories intersection employing FDR adjusted p-values and unbiased empirical distribution as enrichment analysis method) did not result in a further significant KEGG pathways or Gene Ontology processes enrichment of either predicted (TargetScan) or known (Tarbase) target mRNAs (data not shown^24^). Our findings align with previous observations indicating that miRNA changes perform better than mRNA changes in the classification/diagnosis of tumors^10^. So, the concept of persistent radiation-related gene expression changes may provide an avenue for early prediction and diagnosis of cancer which, again, has to be proven in future studies.

Long term sequelae of radiation-related or cancer-associated symptoms such as fatigue, depression and sleep disturbances after radiation treatment or chemotherapy have been well documented by others^15,17^. Our study supports the notion that if irradiation of healthy animals produces persistent molecular changes then this might represent the molecular basis for symptoms persisting over months and years after exposure^5,16^. Several of our candidate genes are known to impact immunological and inflammatory mechanisms (e.g. miR-130b, miR-29c, miR-720, miR-151a-5p, miR-195, miR-212). This observation is in line with a recent publication where another group showed a persistent altered state of the immune system (based on gene expression measurements) up to 30 days after whole thorax irradiation in non-human primates^25^. Noteworthy, immunological alterations have been implicated as a mechanism for persistent fatigue after treatment^18,19^, (supplement Table 1). In our experiment we saw no dramatic changes in animal behavior or activity which could be caused by fatigue. However, a transient body weight loss was observed in three baboons. Clearly, this endpoint (fatigue) was not the main focus of this experiment so that we cannot exclude an unobserved milder grade fatigue.

Interestingly miR-212, which was persistently 48-78-fold downregulated over the entire time period, is already known for its impact on radiosensitivity of glioma cells by targeting *BRCA1*, its immune modulating effect on primary human natural killer cells through IL-12 and its association with breast and prostate cancer, making it a particularly interesting target for further clinical investigation (supplement Table 1).

Some limitations of our study should be kept in mind. We performed gene expression measurements on microarrays and qRT-PCR using human genomic sequences because the baboon transcriptome was not publically available. Given the high homology of both genomes (93%) and previously reports by other groups^26–28^, we proceeded as described above. Since we used TaqMan chemistry with human primer and probe sequences (high sensitivity and specificity), it is more likely that we lost some of the detectable human RNA species due to a mismatch with the baboon genome, rather than producing false positives. Also, in validation studies using human samples from irradiated patients, we were able to reproduce candidate genes from our previous baboon studies implying that differences in genomic sequences represent a minor problem^29^.

The small sample size of our study represents a weakness and surely reduced the number of successfully validated candidate genes. Future work should consider larger sample sizes and results should be validated on additional species.

As a better study design it would be preferable performing longitudinal studies in patients and taking biosamples at several time points during weeks and months of follow-up. However, examining this mechanism of radiation-related persistent gene alterations in cancer patients is not possible, because some therapeutic regimens (e.g. bone marrow transplant) will obscure the radiation-related gene expression changes we detected in our healthy baboons. This on the other hand emphasizes the significance of our results.

Finally, we are very much aware about the speculative nature of the clinical implications of our findings, since we neither measured secondary tumors nor sequela such as fatigue in our baboons. What we are trying here is to put our results into a context of clinical implications and to provide possible hints on underlying molecular mechanisms to be examined in future studies.

In summary, weeks and months after radiation exposure we identified substantial and persistent changes in the post-transcriptional level of 21 miRNAs with known associations to tumorigenesis, radiosensitivity and immune response. We speculate whether this might provide the molecular basis for the development of secondary tumors and a radiation-related genesis of persisting behavioral symptoms such as fatigue observed over months and years after cancer treatment.

## Electronic supplementary material

Supplement figure 1 + Supplement table 1

Supplement table 2

## Data Availability

The datasets generated during or analyzed during the current study are available from the corresponding author on reasonable request.
