# Supplementary material for: Persistent mRNA and miRNA expression changes in irradiated baboons
Source: Sci Rep. 2018 Oct 18;8:15353. doi: 10.1038/s41598-018-33544-2 (PMC6194144; doi:10.1038/s41598-018-33544-2)
Supplement: Supplementary file 1 — Supplement figure 1 + Supplement table 1 [file 41598_2018_33544_MOESM1_ESM.pdf]

Persistent mRNA and miRNA expression changes in irradiated baboons

Port M[1], Herodin F[2], Valente M[2], Drouet M[2], Ostheim P[1], Majewski M[1], Abend M[1,*]

[1]Bundeswehr Institute of Radiobiology, Munich Germany
[2]Institut de Recherche Biomedicale des Armees, Bretigny-sur-Orge, France

REVISED Version
submitted to
Scientific Reports
August 2018

Number of figures: 2 (+1 supplement)
Number of tables: 3 (+1 supplement)

* Corresponding Author: Michael Abend,
Bundeswehr Institute of Radiobiology affiliated to the University of Ulm,
Neuherbergstr. 11, 80937 Munich, Germany, phone/fax: +49-89-992692-2280/-2295,
e-mail: michaelabend@bundeswehr.org

This work was supported by both the French and the German Ministry of Defense

Supplement figure 1

The flow diagram depicts the included samples, split study design, gene expression measurements and statistical analysis.

Supplement table 1

Gene description and annotation (Gene ID, gene alias and chromosome location/sequence) of miRNAs (from table 3) are provided. A literature search (PubMed database/central, Feburary 2018) examined the function of these miRNA as well as their association with tumorigenesis, immune response and radiation exposure. We employed mirBase (mirbase.org, January 2018) and NCBI Nucleotide database (ncbi.nlm.nih.gov/nucleotide) for gene annotations. The actual miRNA ID according to the mirBase was identified based on the deposited miRNA sequence of the LDA. The miR-720 probably represents a fragment of a tRNA [Schopman NCT, Heynen S, Haasnoot J, Berkhout B (2010) A miRNA-tRNA mix-up: tRNA origin of proposed miRNA. RNA Biol. 7:573–576].

Supplement figure 1

Port et al. Persistent mRNA and miRNA expression changes in irradiated baboons.

Stage I (screening)

1. random split sample set: stage I, n=26 / stage II, n=42
2. screening set with
   - 5 RNA samples taken before irradiation (unexposed, pre-exposure samples)
   - 3x7 RNA samples from days 7, 28 & 75-106 after irradiation, respectively
3. mRNA transcriptome (whole genome microarray, 19,596 genes)
   - Filter: Fold change (reference: pre-exposure sample) $\geq |2|$, $p<0.05$, expressed $\geq$ 60%
   - 324 up-regulated and 366 down-regulated genes over all time points
   - < 10 genes overlapping over all time points
   - 2-3 fold decrease in differential expressed genes over time
   - Selection criteria: fold-change height, persistency over time

   → **32 mRNA for stage II**
4. miRNA post-transcriptome (667 miRNAs)
   - 290 miRNAs eligible for analysis
   - Filter: Fold change (reference: pre-exposure sample) $\geq |2|$, $p<0.05$, expressed > 60%
   - Selection criteria: fold-change height, persistency over time

   → **70 miRNA for stage II**

Stage II (validation)

1. independent validation set with 42 samples
   a. mRNA: additional methodological change (from microarry to qRT-PCR)
   b. miRNA: only independent validation on other samples, but employing qRT-PCR
2. duplicate measurement of 32 mRNAs and single measurements of 70 miRNAs using qRT-PCR
3. descriptive statistic of candidate genes
4. Testing for normality and equal variance
5. Examining for group differences using t-test, Kruskal-Wallis test (where appropriate) and logistic regression analysis and ROC curve to quantify the discrimination of pre-exposure group versus the radiation group per time point and candidate mRNA and miRNA
6. Bonferroni correction of p-values for multiple comparisons

→ **validated genes: 2 mRNAs; 21 miRNAs**

supplemental table 1

Port et al. Persistent mRNA and miRNA expression changes in irradiated baboons.

miRBase			literature search	
**Gene ID**	**Gene Alias**	**sequence**	**effect**	**reference**
hsa-miR-378a	miR-378, MIR378, MIRN378, hsa-mir-378, miRNA378, miR378a, MIR378a, MIRN378a, hsa-mir-378a, miRNA378a	AGGGCUCCUGACUCCAGGUCCUGUGUGUUACCUAGAAAUAGCACUGGACUUGGAGUCAGAAGGCCU	**Function / tumor associated**	
			• miR-378a expression might be considered as novel biomarkers to predict the efficacy of vaccine treatment against colorectal cancer	Tanaka et al., Cancer Sci. 2017
			• aberrant expression of miR-124-3p, miR-129-5p and miR-378 in primary tumors, downregulation of hsa-miR-378 induced an increase in both expression and activity of CPT1A, CACT and CrAT in malignant prostate cells	Valentino et al., Oncogene 2017
			• miR-378 inhibits the proliferation, migration and invasion of colon cancer cells	Zeng et al., Cell Mol Biol Lett. 2017
			• miR-378 transfection enhanced cell survival, tumor growth, and angiogenesis through repression of the expression of two tumor suppressors	Lee et al., Proc. Natl. Acad. Sci USA 2007
			• miR-378 is overexpressed in ovarian cancer cells and tumors vs. normal ovarian epithelial cells	Chan et al., Gynecol Oncol. 2014
			• miRNA-378 was increased in serum of patients with renal cell carcinoma compared to healthy controls	Redova et al. J Transl Med. 2012
			**Immune response**	
			• adipokines induced miR-378 expression and revealed the most likely mechanism of adipokine-induced miR-378 dysregulation in human adipocytes. miRNAs have been shown to function in regulating obesity-related metabolic syndrome, and miR-378 may be a novel target for controlling adipose tissue inflammation	Jiang et al. Cell Physiol Biochem. 2014
			**Radiation-induced**	
			• up-regulated in serum of macaques 24h after irradiation (5.8 Gy (LD30/60), 6.5 Gy (LD50/60), and 7.2 Gy TBI)	Fendler et al. Sci Transl Med. 2017
			• up-regulated with highest level of induction at the 8-h time-point in TK6 cells after irradiation and a decline at 4h-, 12h- and 24h timepoints (X-ray 2 Gy)	Chaudhry et al. J Radiat Res. 2013
			• in vitro, miR-378 expression moderately increased cell growth rate and plating efficiency, but did not alter radiation sensitivity; in vivo, miR-378 expression improved tumor oxygenation, thereby increasing the tumor local radiation response and the survival time of irradiated brain tumor-bearing hosts	Wende et al. J Neurooncol. 2018
hsa-miR-22	miR-22, MIR22, MIRN22, hsa-mir-22, miRNA22	GGCUGAGCCGCAGUAGUUCUUCAGUGGCAAGCUUUAUGUCCUGACCCAGCUAAAGCUGCCAGUUGAAGAACUGUUGCCCUCUGCC	**Function / tumor associated**	
			• miR-22 suppresses tumorigenesis and improves radiosensitivity of breast cancer cells	Zhang et al. Biol. Res. 2017
			**Immune response**	
			• regulation of inflammation and apoptosis insubarachnoid hemorrhage	Yu et al. Int J Mol Med. 2018
			**Radiation-induced**	
			• up-regulated at the 8-h and 24h time-points in TK6 cells after irradiation and a decline at 4h-, 12h- and 24h timepoints (X-ray 2 Gy)	Chaudhry et al. J Radiat Res. 2013
hsa-miR-584	miR-584, MIR584, MIRN584, hsa-mir-584, miRNA584	UAGGGUGACCAGCCAUUAUGGUUUGCCUGGGACUGAGGAAUUUGCUGGGAUAUGUCAGUUCCAGGCCAACCAGGCUGGUUGGUCUCCCUGAAGCAAC	**Function / tumor associated**	
			• microRNA-584 inhibits the colorectal cancer progression	Konishi et al. Biochem Biophys Res Commun. 2015
			• miR-584 Suppresses Invasion and Cell Migration of Thyroid Carcinoma	Xiang et al. Oncol Res Treat. 2015
			**Immune response**	
			• significantly dysregulatedin relapsing-remitting MS (RRMS) patients compared with healthy controls	Keller et al. PLOS ONE. 2009
			• significantly up-regulated in inflamed pulps as compared with normal pulps	Zhong et al. J Endod. 2012
hsa-miR-660	miR-660, MIR660, MIRN660, hsa-mir-660, miRNA660	CUGCUCCUUCUCCCAUACCCAUUGCAUAUCGGAGUUGUGAAUUCUCAAAACACCUCCUGUGUGCAUGGAUUACAGGAGGGUGAGCCUUGUCAUCGUG	**Function / tumor associated**	
			• downregulated in lung cancer patients and its replacement inhibits lung tumorigenesis	Fortunato O et al. Cell Death Dis. 2014
			**Radiation-induced**	
			• up-regulated at the 8-h and 24h time-points inhuman fibroblasts after irradiation (X-ray 2 Gy)	Maes et al. J. Cell. Biochem 2015
hsa-miR-324-5p	miR-324-5p, MIR324-5p, MIRN324-5p, hsa-mir-324-5p, miRNA324-5p	CGCAUCCCCUAGGGCAUUGGUGU	**Function / tumor associated**	
			• miR-324-5p inhibits proliferation of glioma	Xu et al. Eur Rev Med Pharmacol Sci. 2014
hsa-miR-664a	miR-664, MIR664, MIRN664, hsa-mir-664, miRNA664, miR-664a, MIR664a, MIRN664a, hsa-mir-664a, miRNA664a	GAACAUUGAAACUGGCUAGGGAAAAUGAUUGGAUAGAAACUAUUAUUCUAUUCAUUUAUCCCCAGCCUACAAAAUGAAAAAA	**Function / tumor associated**	
			• upregulated expression of miR-664a could have an inhibitory effect on MEG3 gene expression and migration of osteosarcoma cells	Sahin et al. Biochem Biophys Res Commun. 2017
			• overexpression of miR-664 is associated with enhanced osteosarcoma cell migration and invasion ability	Boa et al. Clin Exp Med. 2017
			• miR-664 negatively regulates PLP2 and promotes cell proliferation and invasion in T-cell acute lymphoblastic leukaemia	Zhu et al. Biochem Biophys Res Commun. 2015
			**Immune response**	
			• significantly dysregulatedin relapsing-remitting MS (RRMS) patients compared with healthy controls	Keller et al. PLOS ONE. 2009
RNU48	SNORD48, U48	AGUGAUGAUGACCCCAGGUAACUCUGAGUGUGUCGCUGAUGCCAUCACCGCAGCGCUCUGACC	**Radiation-induced**	
			• high stability of expression following irradiation in irradiated prostate cancer cells (x-ray single 6-Gy radiation dose)	Lawlor et al. Tumour Biol. 2015
hsa-miR-1305	miR-1305, MIR1305, MIRN1305, hsa-mir-1305, miRNA1305	AAGAUCCUGCUGUUUCUACCAUUAGUUUUGAAUGUUUAUUGUAAAGAUACUUUUCAACUCUAAUGGGAGAGACAGCAGGAUUCUCC		
hsa-miR-130b	miR-130b, MIR130b, MIRN130B, mir-130b, hsa-mir-130b, miRNA130b	GGCCUGCCCGACACUCUUUCCCUGUUGCACUACUAUAGGCCGCUGGGAAGCAGUGCAAUGAUGAAAGGGCAUCGGUCAGGUC	**Function / tumor associated**	
			• up-regulation promotes lung cancer progression	Jianwei et al. J Exp Clin Cancer Res. 2016
			• miR-130 family promotes cell migration and invasion in bladder cancer	Hiroshi et al. Sci Rep. 2016
			**Immune response**	
			• attenuates vascular inflammation via negatively regulating tumor progression locus 2 (Tpl2) expression	Wang et al. Int Immunopharmacol. 2017
			• silencing of H19 inhibits the adipogenesis and inflammationresponse in ox-LDL-treated Raw264.7 cells by up-regulating miR-130b	Han et al. Mol Immunol. 2018
			• inflammation-associated microRNA-130b down-regulates cytochrome P450 activities and directly targets CYP2C9	Rieger et al. Drug Metab Dispos. 2015
			**Radiation-induced**	
			• up-regulated at the 8-h time-point in TK6 cells after irradiation and down-regulated at 4h-, 12h- and 24h timepoints (X-ray 2 Gy)	Chaudhry et al. J Radiat Res. 2013
hsa-miR-500-a	miR-500a, MIR500a, MIRN500a, mir-500a, hsa-mir-500a, miRNA500a, miR-500,	GCUCCCCCUCUCUAAUCCUUGCUACCUGGGUGAGAGUGCUGUCUGAAUGCAAUGCACCUGGGCAAGGAUUCUGAGAGCGAGAGC	**Function / tumor associated**	
			• potential diagnostic marker for hepatocellular carcinoma	Yamamoto et al. Biomarkers. 2009
			• upregulated in both prostate cancer cells and primary tumors	Cai et al. Prostate. 2017
hsa-miR-151a-5P	miR-1515p, MIR1515p, MIRN1515p, mir-151a-5P, hsa-mir-151a-5p, miRNA1515P	UCGAGGAGCUCACAGUCUAGU	**Function / tumor associated**	
			• up-regulated in patients with lung adenocarcinomas	Cazzoli et al. J Thorac Oncol. 2013
			• represses Migration of Human Breast Cancer Cells	Yeh et al. PLoS One. 2016
			• enhance migration and metastasis in human hepatocellular carcinoma (HCC)	Ding et al. Nat Cell Biol. 2010
			**Immune response**	
			• downregulated in fibromyalgia	Bjersing et al. Rheumatol Int. 2015
			• directly or indirectly influences humoral immunity to measles vaccination as a B cell-specific and suggests that these miRNAs may serve as useful predictive biomarkers of vaccine humoral immune response	Haralambieva et al. PLoS One. 2018
			**Radiation-induced**	
			• up-regulated approximately 3-fold in squamous cell carcinoma of the head and neck and cell lines from brain tumors (2Gy Photons)	Niemoeller et al. Radiat Oncol. 2011
hsa-miR-29c	miR-29c, MIR29C, MIRN29C, hsa-mir-29c, miRNA29c	AUCUCUUACACAGGCUGACCGAUUUCUCCUGGUGUUCAGAGUCUGUUUUUGUCUAGCACCAUUUGAAAUCGGUUAUGAUGUAGGGGGA	**Function / tumor associated**	
			• regulates radioresistance in lung cancer cells as a tumor suppressor	Arechaga-Ocampo et al. Tumour Biol. 2017
			**Immune response**	
			• TGF-beta/Smad3 signaling promotes renal fibrosis by inhibiting miR-29	Quin et al. J Am Soc Nephrol. 2011
			• miR-29c was significantly increased in the serum of early stage NSCLC cases	Heegaard et al. Int J Cancer. 2012
			• role of miR-29 in IFN-γ regulation in NK cells	Ma et al. Nat Immunol. 2011
			• MiRNA-29: a microRNA family with tumor-suppressing and immune-modulating properties	Schmitt et al. Curr Mol Med. 2013
			• important functions of the miR-29 family have been uncovered, identifying miR-29 as a crucial regulator of thymic function, T cell polarization and B cell oncogenesis	Liston et al. Cell Mol Life Sci. 2012
			• miRNA-29 family could influence the clinical progression of HIV-1 infection, the HIV-1 proviral load and the innate immune response against HIV-1	Monteleone et al. BMC Infect Dis. 2015
			• serum microRNA-29 levels correlate with disease progression in patients with chronic hepatitis B virus infection	Huang et al. J Dig Dis. 2014
			• essential roles of specific microRNAs in regulating immunefunction of CB cells	Takahashi et al. Eur J Haematol. 2012
			**Radiation-induced**	
			• upregulated in primary human dermal microvascular endothelial cells (HDMEC) after ionizing radiation (2 Gy)	Wagner-Ecker et al. Radiat Oncol. 2010
			• greatly downregulated in the FS/Control group (10- to 50-fold) but slightly upregulated in the FS/radiation group in mice lung (single-fraction 13.5 Gythoracic X-ray radiation)	Christofidou-Solomidou et al. Cancer Biol Ther. 2014
			• low-dose ionizing radiation (IR) induces miR-29c expression in female mouse liver, while inhibiting its expression in a human hepatocellular carcinoma cell line (3Gy)	Wang et al. Oncotarget. 2015
hsa-miR-331-5p	miR-331-5p, MIR3315P, MIRN3315P, mir-331-5p, hsa-mir-331-5p, miRNA3315P	CUAGGUAUGGUCCCAGGGAUCC	**Function / tumor associated**	
			• down-regulated miR-331–5p associated with chemotherapy resistance and relapse in leukaemia	Feng et al. J Cell Mol Med. 2011
hsa-miR-532-3p	miR-532-3p, MIR5323P, MIRN5323P, mir-532-3p, hsa-mir-532-3p, miRNA5323P	CCUCCCACACCCAAGGCUUGCA	**Function / tumor associated**	
			• up-regulated in triple-negative breast tumors	Li et al. PLoS One 2013
			• inhibits cell proliferation and metastasis by targeting the CXCL2 oncogene in hepatocellular carcinoma	Song et al. Am J Transl Res. 2015
			• mir-532-3p promotes cell growth, migration and invasion in gastric cancer cells by down-regulating RUNX3	Xu et al. J Cell Mol Med. 2016
			• mir 532-3p up-regulated in melanoma lines and metastatic melanoma tumors which promotes cell growth, migration and invasion in melanoma by down-regulating RUNX3	Kitago et al. Clin Cancer Res. 2009
			**Immune response**	
			• downregulated in liposaccharide-induced RAW264.7 macrophage cells	Cheng et al. Inflammation 2012
hsa-miR-195	miR-195, MIR195, MIRN195, mir-195, hsa-mir-195, miRNA195	AGCUUCCCUGGCUCUAGCAGCACAGAAAUAUUGGCACAGGGAAGCGAGUCUGCCAAUAUUGGCUGUGCUGCUCCAGGCAGGGUGGUG	**Function / tumor associated**	
			• suppresses the metastasis and epithelial-mesenchymal transition of hepatocellular carcinoma by inhibiting YAP	Yu et al. Oncotarget. 2017
			• upregulation of miR-195 enhances the radiosensitivity of breast cancer cells through inhibition of BCL-2	Zhu et al. Int J Clin Exp Med. 2015
			• inhibits tumor growth and metastasis in papillary thyroid carcinoma cell lines by targeting CCND1 and FGF2	Yin et al. Int J Endocrinol. 2017
			**Immune response**	
			• inhibits macrophages pro-inflammatory profile and impacts the crosstalk with smooth muscle cells --> might be used as a new potential tool to promote inflammation resolution in cardiovascular research	Bras et al. PLoS One. 2017
			**Radiation-induced**	
			• enhances radiosensitivity of colorectal cancer cells through suppressing CARM1. Therefore, miR-195 acts as a potential regulator of radioresistance for CRC cells (0, 2, 4, 6, and 8 Gy of X-rays)	Zheng et al. Onco Targets Ther. 2017
hsa-miR-422a	miR-422a, MIR422a, MIRN422a, mir-422a, hsa-mir-422a, miRNA422a	GAGAGAAGCACUGGACUUAGGGUCAGAAGGCCUGAGUCUCUCUGCUGCAGAUGGGCUCUCUGUCCCUGAGCCAAGCUUUGUCCUCCCUGG	**Function / tumor associated**	
			• up-regulated in osteosarcoma tissues and cell lines; inhibits osteosarcoma proliferation by targeting BCL2L2 and KRAS	Zhang et al. Biosci Rep. 2018
			• down-regulated in CRC tissues and cell lines; inhibits cell proliferation in colorectal cancer by targeting AKT1 and MAPK1	Wie et al. Cancer Cell Int. 2017
			• inhibits glioma proliferation and invasion by targeting IGF1 and IGF1R	Wang et al. Oncol Res. 2017
hsa-miR-212	miR-212, MIR212, MIRN212, mir-212, hsa-mir-212, miRNA212	CGGGGCACCCCGCCCGGACAGCGCGCCGGCACCUUGGCUCUAGACUGCUUACUGCCCGGGCCGCCCUCAGUAACAGUCUCCAGUCACGGCCACCGACGCCUGGCCCCGCC	**Function / tumor associated**	
			• inhibitory effect on HCC by inhibiting FOXA1 expression	Dou et al. Oncotarget. 2015
			• modulates the radiosensitivity of glioma cells by targeting BRCA1	He et al. Oncol Rep. 2017
			**Immune response**	
			• up-regulation during prolonged IL-12 treatment, negatively regulates the IL-12 signaling pathway by reducing STAT4 expression in primary human NK cells	Huang et al. Blood. 2011
hsa-miR-423-5p	miR-423-5p, MIR4235P, MIRN4235P, mir-423-5p, hsa-mir-423-5p, miRNA4235p	UGAGGGGCAGAGAGCGAGACUUU	**Function / tumor associated**	
			• up-regulated in the brain metastasis patients; increased expression as an independent risk factor for BM in lung adenocarcinoma	Sun et al. Int. J. Radiat. Oncol. Biol. Phys. 2017
			**Radiation-induced**	
			• up-regulated with highest level of induction at the 8-h time-point in TK6 cells after irradiation and a decline at 4h-, 12h- and 24h timepoints (X-ray 2 Gy)	Chaudhry et al. J Radiat Res. 2013
hsa-miR-454	miR-454, MIR454, MIRN454, mir-454, hsa-mir-454, miRNA454	UCUGUUUAUCACCAGAUCCUAGAACCCUAUCAAUAUUGUCUCUGCUGUGUAAAUAGUUCUGAGUAGUGCAAUAUUGCUUAUAGGGUUUUGGUGUUUGGAAAGAACAAUGGGCAGG	**Function / tumor associated**	
			• promoted the proliferation of triple negative breast cancer and enhanced migration and invasion in TNBC cells; potential biomarker to predict radiotherapy response	Li et al. J Biol Res (Thessalon). 2017
			**Immune response**	
			• possible biomarker for disease staging in multiple sclerosis	Gandhi et al. Ann Neurol. 2013
			**Radiation-induced**	
			• down-regulated miR-454-3p levels 24, 48 and 72h after irradiation in human renal carcinoma 786-O cells; down-regulation of BTG1 by miR-454-3p enhances cellular radiosensitivity in renal carcinoma cells (5 Gy X-rays)	Wu et al. Radiat Oncol. 2014
hsa-miR-139-5p	miR-139, MIR139, MIRN139, mir-139, hsa-mir-139, miRNA139, miR-139-5p, MIR1395P, MIRN1395P, mir-139-5p, hsa-mir-139-5p, miRNA1395p	UCUACAGUGCACGUGUCUCCAGU	**Function / tumor associated**	
			• treatment of breast cancer cells with a miR-139-5p mimic strongly synergized with radiation both in vitro and in vivo; potentially useful predictive biomarker of radiosensitivity in solid tumors and a generally applicable druggable target for tumor radiosensitization (0 to 12Gy dose fractioned X-Ray)	Pajic et al. Cancer Res. 2018
			• 25-HC inhibited miR-139-5p expression in mouse bone marrow macrophages; 25-hydroxycholesterol promotes osteoclastogenesis through coordinating NFATc1 and Sp1 complex in transcription of miR-139-5p	Zhang et al. Biochem Biophys Res Commun. 2017
			• negatively regulated the proliferation of hematopoietic stem cells and progenitor cells and downregulation of miR-139-5p expression was associated with hematopoietic malignancy, such as chronic myeloid leukemia (CML); repressed during BCR-ABL-mediated leukemogenesis	Choi et al. Blood. 2016
			• inhibits aerobic glycolysis, cell proliferation, migration, and invasion in hepatocellular carcinoma via a reciprocal regulatory interaction with ETS1	Hua et al. Oncogene. 2018
hsa-miR-720	miR-720, MIR720, MIRN720, mir-720, hsa-mir-720, miRNA720	CCGGAUCUCACACGGUGGUGUUAAUAUCUCGCUGGGGCCUCCAAAAUGUUGUGCCCAGGGGUGUUAGAGAAAACACCACACUUUGAGAUGAAUUAAGAGUCCUUUAUUAG	**Function / tumor associated**	
			• down-regulated in NSCLC patient samples, i.e. lowest expression in NSCLC	Leidinger et al. Oncotarget. 2016
			• upregulated in HBV-specific CD8+ T cells. Overexpression in primary human CD8+ T cells inhibits TCR stimulation-induced proliferation. Upregulation after TCR stimulation --> therapies targeting miR-720 may help restore impaired immunity in CHB patients	Wang et al. Sci Rep. 2015
			• down-regulated levels following resection of metastatic melanoma	Latchana et al. Bioinform Biol Insights. 2017
			**Immune response**	
			• plays an important role in regulating M2 macrophage polarization and function	Zhong et al. Biosci Rep. 2016
			**Radiation-induced**	
			• down-regulated after low dose irradiation and even more after high dose irradiation in human-hamster hybrid cells (X-ray 0.5 and 2 Gy)	Aypar et al. Mutat. Res. 2011
